# *In Vitro* Comparison of Different Invisalign® and 3Shape® Attachment Shapes to Control Premolar Rotation

**DOI:** 10.3389/fbioe.2022.840622

**Published:** 2022-03-16

**Authors:** Nikolaos Ferlias, Michel Dalstra, Marie A. Cornelis, Paolo M. Cattaneo

**Affiliations:** ^1^ Section of Orthodontics, Department of Dentistry and Oral Health, Aarhus University, Aarhus, Denmark; ^2^ Melbourne Dental School, Faculty of Medicine, Dentistry and Health Sciences, The University of Melbourne, Melbourne, VIC, Australia

**Keywords:** orthodontics, clear aligners, biomechanics, tooth movement, 3D printing

## Abstract

**Aim:** To evaluate *in vitro* the differences of various Invisalign® attachments in their effectiveness during derotation of an upper second premolar in terms of forces and moments created and compare them to the 3Shape® box attachment as well as to no attachment at all.

**Materials and Methods:** A Force System Identification (FSI) machine, comprising two load sensors, was used in this study. Sensor 1 was connected to the test tooth (i.e. upper second premolar) carrying a different attachment design, and the fixed sensor (Sensor 2) was connected to the base model. Once the corresponding aligner was passively seated on the teeth, 12 different setups (i.e. 11 different attachments and one setup with no attachment at all) were tested by rotating the test tooth 4.5° mesially and 4.5° distally, in increments of 0.45°.

**Results:** The vertical rectangular attachments were able to generate the highest derotational moment on both mesial and distal rotations but also received the most side effects (intrusive force, torque, and tipping). The no-attachment setup performed least favorably in terms of derotational ability but exhibited the least side effects. In the *y*-axis, all attachments received a buccal root torque with a lingual force during disto-rotation and a lingual root torque with a buccal force during mesio-rotation.

**Conclusion:** Attachments are necessary for derotating an upper second premolar. An aligner incremental change of more than 1° derotation can generate high moments. The vertical rectangular attachments perform best in derotations; however, they exhibit the most side effects. Finally, despite presenting the least side effects, derotation of a premolar with no attachment is not as efficient.

## Introduction

Clear aligner therapy has been an alternative treatment modality to conventional orthodontic fixed appliances to clinicians for almost 20 years, despite the concept having been introduced almost half a century ago (Kesling, 1945). However, the initial limitations of the aligners’ clinical applications kept the orthodontic community quite skeptical in the beginning. These were most commonly the lack of finishing control with imprecise final detailing and the lack of rotational control and poor aligner fit due to lack of compliance (Sheridan, 2004). In the rapid evolutionary process that followed, orthodontists came to acknowledge clear aligner therapy as a valid alternative to conventional multi-bracket systems. Patients played an important role in this change, as a significant number of them find metal or ceramic fixed appliances unattractive and unacceptable (Rosvall et al., 2009). This evolution, however, may have come with inadequate evidence, with the research community in general not being able to keep up with the aligners’ fast-growing pace, thus leading to a technology bypass.

It is true that compliance is the most important factor in clear aligner therapy, yet retention of the aligner is equally crucial if we want to apply the necessary forces to achieve the desired tooth movements. There are certain movements that prove to be quite challenging in every day clinical practice; these are mainly the torque and root angulation, extrusion as well as rotation, especially of those more rounded teeth, like the canines and premolars (Rossini et al., 2015; Bowman, 2017). In fact, Kravitz et al. (Kravitz et al., 2009) report that derotation of a lower canine is the least accurate movement when compared to all other teeth. In addition, the mean accuracy of tooth movement with clear aligners is as low as 41% with the extrusion being the least accurate (Kravitz et al., 2009).

The company that created the market, Invisalign® (Align Technology, San Jose, CA, USA), developed rapidly since its appearance 2 decades ago and added attachments and auxiliaries in order to improve aligner retention and achieve more complex orthodontic tooth movements, in an attempt to apply bio-mechanical principles. Different attachment shapes have been introduced since by Invisalign® and are being used in almost all aligner treatments. These seem to have improved the overall treatment accuracy (Haouili et al., 2020). However, there is lack of evidence regarding the differences in performance between the different types of attachments in achieving certain kinds of orthodontic tooth movement (Dasy et al., 2015). As a consequence, the boundaries between marketing claims and evidence-based clinical reality can be quite blurred.

In a recent retrospective study, the efficacy of different Invisalign® attachments was investigated and no difference was found between conventional and optimized attachment designs (Karras et al., 2021). The movement investigated was rotation of canines and premolars as well as extrusion of anterior teeth which is more challenging to achieve predictably with Invisalign® (Papadimitriou et al., 2018). With the present evolution of 3D printing and the rapid development of in-house aligners, investigating the clinical differences of various attachment shapes can have implications for the clinician in their treatment approach and efficacy.

Therefore, the aim of this study was to evaluate, *in vitro*, the differences of various Invisalign® attachment designs in their effectiveness to derotate of an upper second premolar in terms of forces and moments transmitted to the tooth, and compare those to the 3Shape® box attachment (3Shape®, Copenhagen, Denmark) as well as when no attachment at all is used.

## Materials and Methods

### Design and 3D Printing

**FIGURE 1:**
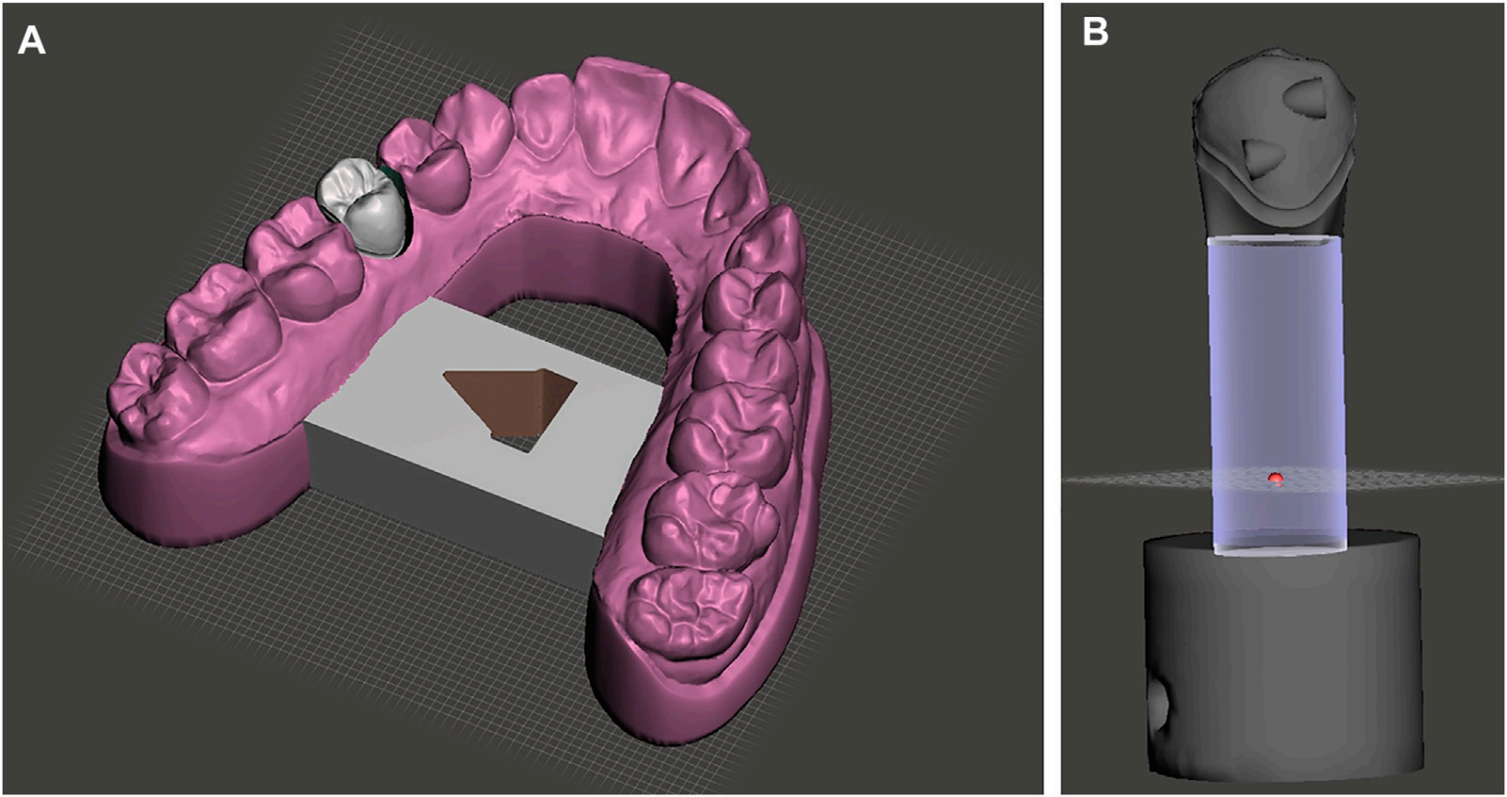
3D design of the set up. **(A)**. Model base used for all tests (in purple) and the test tooth 15 fitted with the various attachments (grey). Please, note the clearance around test tooth 15, which allowed for free rotation around the tooth long axis, without any interference. **(B)**. All the setups of tooth 15 with the different types of attachments were built on the same base tooth model and had the same cylindrical base. Here, the ElliPair attachment (consisting of two hemi-elliptical attachments, HemiElliR and HemiElliL) are depicted.

First, an intraoral scan of an upper jaw with fairly aligned teeth was taken using the TRIOS intraoral scanner by 3Shape® (Copenhagen, Denmark). The various attachments (full description in the next paragraph) were then virtually placed in the middle of the crown (middle of the mesio-distal and apico-occlusal dimensions) using Orthoanalyzer® (3Shape®). The STL files created were then imported into the Dental System® (3Shape®). A new order was then created for a single coping using the Model Builder® workflow. Then, the upper right second premolar with the particular attachment was separated as a single die, and both parts, the model and the die, were then saved and exported as STL files. Finally, these files were imported into Meshmixer (Autodesk®, San Rafael, CA, United States) for the final preparation so that the die and the model could be mounted on the FSI sensors. During this preparation, the same cylindrical base parallel to the long axis of the tooth and with the same dimensions was used for all the different attachment setups (Figure 1).

As far as the 3D printing procedure is concerned, all models as well as the single premolar teeth with the various attachments were printed in a Nextdent 5100® 3D printer (Vertex-Dental, Soesterberg, Netherlands) using the Model 2.0® printing material produced by the same company. The aligners were manually fabricated once all the 12 initial models were printed using the Taglus® aligner foils (Laxmi Dental, Mumbai, India) with a thickness of 0.762 mm.

### Setup

**FIGURE 2:**
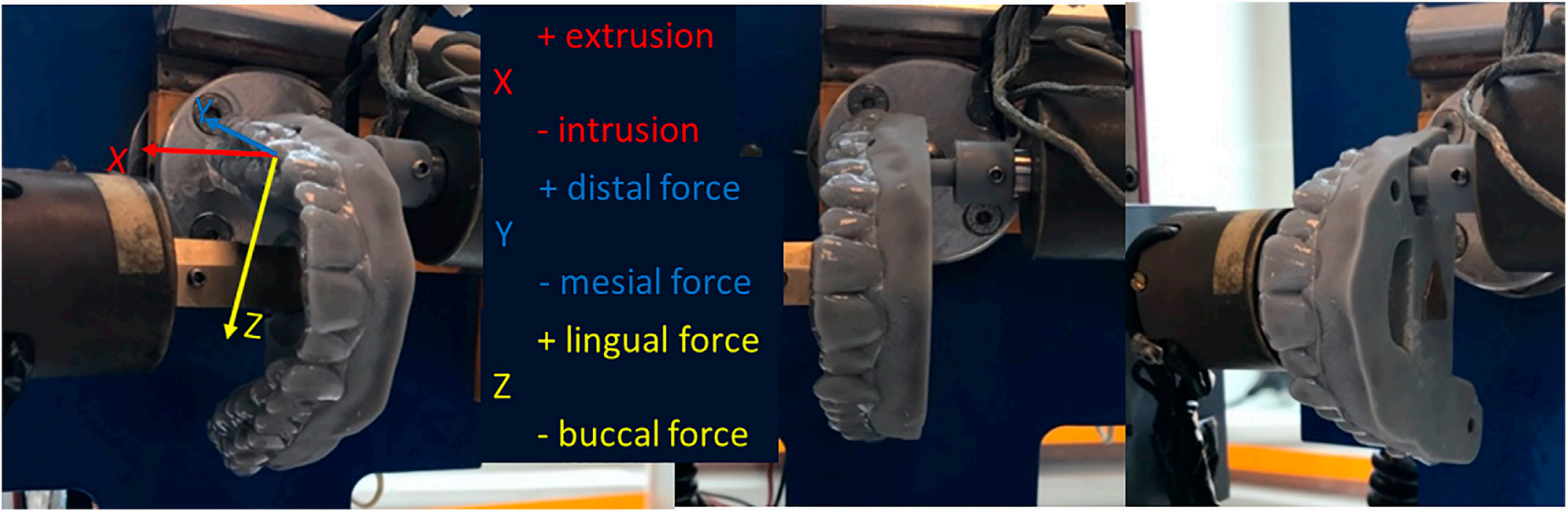
Overview of the testing set-up. Sensor 1 (rotating tooth carrying different attachment design, seen on the right) and Sensor 2 (stable base model, left) in action. Each aligner corresponding to the specific attachment was mounted in the “test position” which was reproduced for all tests by the same FSI software coordinates. The three axes: X: long axis of the tooth; Y: mesio-distal axis; and Z: bucco-lingual axis are represented for intelligibility.

**FIGURE 3:**
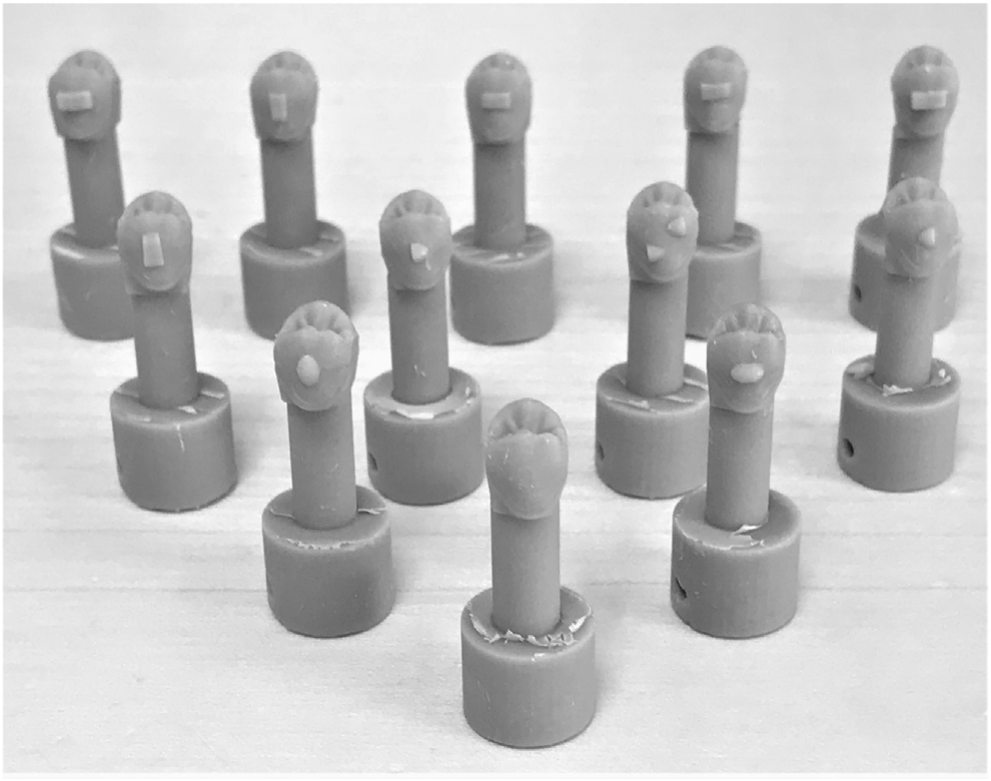
Twelve different setups were tested consisting of eleven Invisalign® attachments, the 3Shape® Box attachment and one setup with no attachment at all serving as control.

**FIGURE 4:**
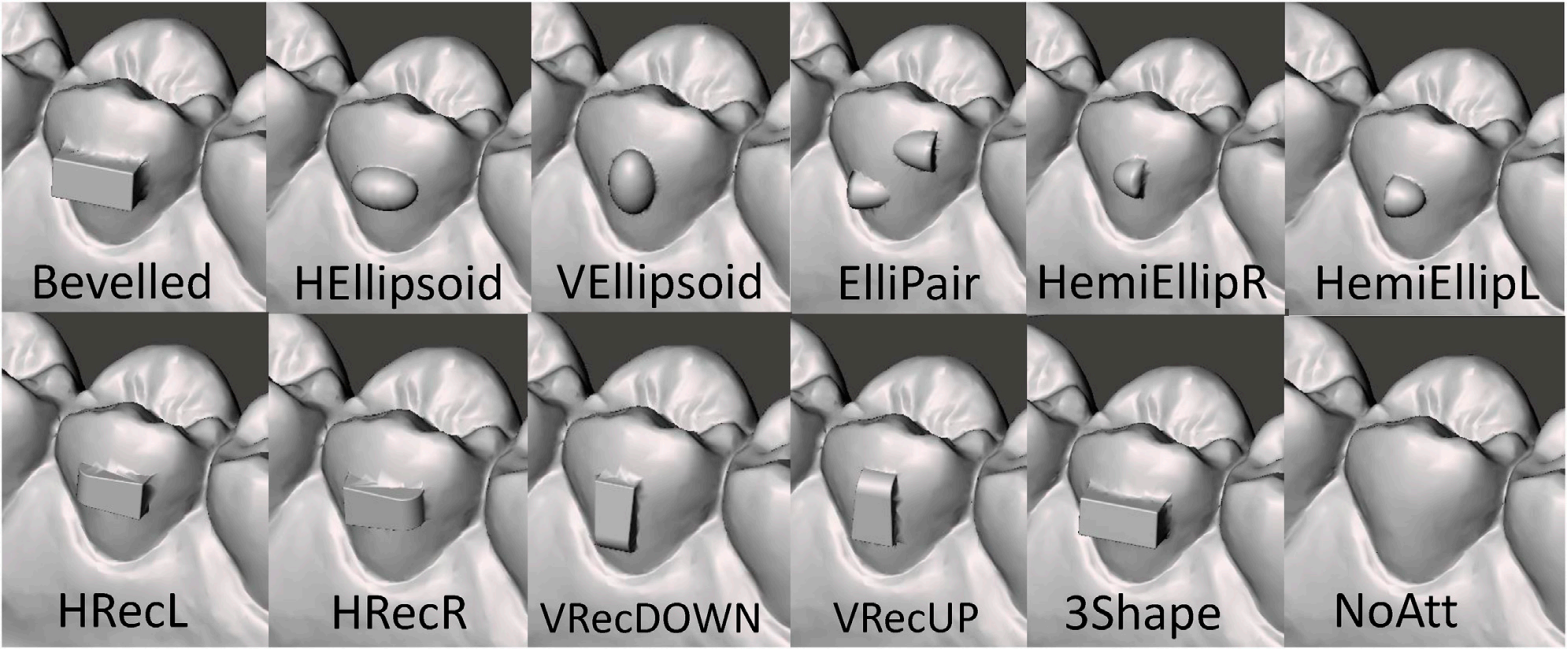
Details of the attachments reported in Figure 3 are presented with the names 411 used throughout the article.

The setup of this study consisted of a dental model that was divided into two separate parts, each one connected to a sensor measuring the forces and moments (F/M) created, using the Force System Identification (FSI) machine, which was developed for the Department of Orthodontics, Aarhus University, Denmark. The first part in the setup was the base model carrying all the upper teeth except tooth 15, at which place a hole was drilled in the model (Figures 1, 2). This part was attached to Sensor 2 of the FSI machine and was kept fixed throughout the whole testing procedure. The second part, connected to Sensor 1 of the FSI machine, was tooth 15 carrying the different attachment at each test. The tooth 15 part was replicated 12 times, each one characterized by 11 different types of attachment and one where no attachment was present. The attachments tested (abbreviation and dimensions in parenthesis) were the Invisalign® “Bevelled” (Bevelled, 3.5 × 1.5 × 1 mm), “Horizontal Ellipsoid” (HEllipsoid, 3 × 2 × 1 mm), “Vertical Ellipsoid” (VEllipsoid 3 × 2 × 1 mm), “Elliptical Pair” (ElliPair, 2 × 2 × 1 mm/each), “Hemi-elliptical Right” (HemiEllipR, 2 × 2 × 1 mm), “Hemi-elliptical Left” (HemiEllipL, 2 × 2 × 1 mm), “Horizontal Rectangular Left” (HRecL, 3.5 × 1.5 × 1 mm), “Horizontal Rectangular Right” (HRecR, 3.5 × 1.5 × 1 mm), “Vertical Rectangular Down” (VRecDOWN, 3.5 × 1.5 × 1 mm), and “Vertical Rectangular Up” (VRecUp, 3.5 × 1.5 × 1 mm). In addition, the regular “3Shape® Box” (3Shape, 3.5 × 1.5 × 1 mm) attachment was also tested, as well as a tooth 15 with “No Attachment” (NoAtt) at all (Figures 3, 4).

The mounting was completed on a reproducible position with saved coordinates (neutral position) and controlled by the FSI computer software.

Once both parts were mounted on the sensors, position two (test position) was reproduced using the FSI’s “test-position” coordinates. The specific aligner corresponding to the particular attachment was then placed on the teeth. Prior to placement, the inside of the aligner was lubricated with a saliva substitute using a squirt on every tooth (GUM® Hydral®, Etoy Switzerland). Then, Sensor 1 was automatically rotated, in steps of 0.45°, up to 4.5° mesial and back to neutral position. Prior to the distal rotation, the aligner was removed and seated again on the teeth and then it was rotated the other direction, 4.5° distal and back to neutral again (Figure 2).

For descriptive reasons, the attachments were arranged in five main groups according to their orientation and shape. Group 1, “Rectangular Vertical” included two rectangular shape attachments with a vertical orientation (i.e. “VRecUp” and “VRecDOWN”). Group 2, “Rectangular Horizontal” included four rectangular shape attachments with a horizontal orientation (i.e. “HRecR”; “HRecL”; “3Shape”; “Bevelled”). Group 3 included one ellipsoid attachment with a vertical orientation (i.e. the “VEllipsoid”). Group 4 consisted of four ellipsoid attachments with a horizontal orientation (i.e., “HEllipsoid”; “HemiEllipR”; “HemiEllipL”). Finally, Group 5 was comprised of twin semi-ellipsoid attachment (i.e., “ElliPair”) and the no-attachment configuration (i.e., “NoAtt”).

In our setup, the *x*-axis coincided with the long axis of the tooth with positive force values (F_x_) corresponding to “extrusion” and negative values corresponding to “intrusion” (Figure 2; Table 1). Similarly, positive values in *x*-axis moments (M_x_) indicated “distal rotation,” while negative values indicated “mesial rotation.” The *y*-axis was the mesio-distal axis, with positive force (F_y_) values indicating “distal” direction and negative values indicating “mesial” direction. When the moments in the *y*-axis (M_y_) were positive, they corresponded to “lingual root torque” and negative to “buccal root torque.” Finally, the *z*-axis was the bucco-lingual axis, where positive values represented the “lingual” direction and the “negative” values a “buccal” force direction (F_z_). The moments in the *z*-axis (M_z_) indicated “distal tipping” when positive and “mesial tipping” when negative.

**TABLE 1:** Forces (F, in cN) and moments (M, in cNmm) at the final positions of 4.5 mesial and 4.5 distal rotations. When the test tooth is 4.5 mesial rotated, forces and moments are created to disto-rotate the tooth (Mx positive).

	Attachment	*x*-Axis				*y*-Axis				*z*-Axis			
		Fx		Mx		Fy		My		Fz		Mz	
		+ (extrusion) − (Intrusion)		+ (distal rotation) − (mesial rotation)		+ (distal) − (Mesial)		+ (lingual root torque) //− (buccal root torque)		+ (lingual) − (Buccal)		+ (distal tipping) − (Mesial tipping)	
Tooth Rotation		4.5° Mesial	4.5° Distal	4.5° Mesial	4.5° Distal	4.5° Mesial	4.5° Distal	4.5° Mesial	4.5° Distal	4.5° Mesial	4.5° Distal	4.5° Mesial	4.5° Distal
Group 1: “Rectangular Vertical”	VRecUp	−170	−70	7,640	−9,218	27	−151	−9,627	7,497	330	−255	2,662	−4,020
	VRecDOWN	−159	−40	6,761	−9,221	−5	−216	−7,546	7,704	266	−264	1,476	−5,402
Group 2: “Rectangular Horizontal”	HRecR	−165	3	6,560	−6,965	21	−136	−6,641	5,118	224	−176	1941	−4,645
	HRecL	−75	−79	3,857	−5,200	24	−86	−5,298	2,956	176	−93	1862	−2,438
	3Shape®Box	−177	52	5,680	−3,680	44	−67	−4,575	3,250	109	−78	1897	−2,274
	Bevelled	−118	−38	2,584	−6,827	22	−93	−1,083	3,911	37	−145	1,208	−2,392
Group 3: “Ellipsoid Vertical”	Vellipsoid	−147	−36	5,515	−5,245	73	−117	−5,948	5,880	193	−184	2,762	−3,434
Group 4: “Ellipsoid Horizontal”	Hellipsoid	−122	−9	4,850	−1956	54	−40	−4,578	1909	165	−63	2,527	−1,307
	HemiEllipR	−135	28	4,674	−3,351	56	−20	−3,378	3,002	110	−113	2,324	−945
	HemiEllipL	−170	23	7,861	−5,358	69	−116	−8,116	5,245	301	−184	3,357	−3,500
Group 5: “Other”	ElliPair	−98	−27	5,195	−8,456	29	−173	−5,233	6,520	184	−229	1,643	−4,424
	NoAtt	−65	−137	2,198	−1,582	64	−10	−2042	1970	67	−60	2,363	−96

### Statistics

All measurements were repeated after a minimum of 2 week interval between them. For the error of the method, the intraclass correlation coefficient (ICC) was used to compare the two rounds of measurements. The number of attachments used in the present study was determined purely on the availability of the most commonly used attachment designs.

## Results

The ICC showed excellent reliability (>0.91) for all measurements, except for the HemiEllipR and HEllipsoid, where the ICC was 0.83 and 0.81, respectively.

During the measurements, complete disengagement of the aligner did not occur, although disengagement to some minor extent occurred with some attachment setups. All results for the forces and moments at the end positions (4.5° mesial and 4.5° distal rotation) are summarized in Tables 1, 2 (1.35° mesial and distal rotation).

**TABLE 2:** Forces (F, in cN) and moments (M, in cNmm) in all three axes at 1.35 mesial and 1.35 distal rotations. When the test tooth is 1.35 mesial rotated, forces and moments are created to disto-rotate the tooth (Mx positive).

	Attachment	*x*-Axis				*y*-Axis				*z*-Axis			
		Fx		Mx		Fy		My		Fz		Mz	
		+ (extrusion) /− (intrusion)		+ (distal rotation) /− (mesial rotation)		+ (distal) /− (mesial)		+ (lingual root torque) /− (buccal root torque)		+ (lingual) /− (buccal)		+ (distal tipping) /− (mesial tipping)	
Tooth Rotation		1.35° Mesial	1.35° Distal	1.35° Mesial	1.35° Distal	1.35° Mesial	1.35° Distal	1.35° Mesial	1.35° Distal	1.35° Mesial	1.35° Distal	1.35° Mesial	1.35° Distal
Group 1: “Rectangular Vertical”	VRecUp	−32	−22	2,535	−2,870	4	−15	−2,372	1,212	80	−37	602	−573
	VRecDOWN	−12	−19	1775	−2,984	−22	−53	−693	1,634	19	−57	−281	−1,304
Group 2: “Rectangular Horizontal”	HRecR	−45	18	2,661	−2,507	0	−6	−1,475	955	51	−17	420	−937
	HRecL	2	−10	1,108	−1,354	12	−13	−849	−316	23	14	339	−442
	3Shape®Box	−44	22	2020	−1707	−5	0	−462	38	−17	5	129	−140
	Bevelled	−12	3	646	−2,705	−5	−13	581	461	−23	−16	−88	−278
Group 3: “Ellipsoid Vertical”	Vellipsoid	−22	7	1,254	−1,463	6	−22	−572	972	16	−29	224	−728
Group 4: “Ellipsoid Horizontal”	Hellipsoid	−42	−5	1,623	−819	8	−9	−897	23	36	3	478	−256
	HemiEllipR	−24	35	1779	−1,228	13	5	−575	166	15	−11	504	−99
	HemiEllipL	−48	22	2,594	-1702	13	−19	−1,693	499	64	−19	756	−700
Group 5: “Other”	ElliPair	1	−8	1,560	−2,643	−3	−27	−351	729	11	−26	−23	−731
	NoAtt	−2	−23	828	−862	11	−10	−416	785	14	−24	517	−254
	= highest												
	= lowest												

### *x*-Axis

As far as the *x*-axis is concerned (long axis of the tooth), when 15 was mesially rotated, the attachment producing the highest force was the 3Shape, generating an intrusive force of 177cN. At the other side of the range, the tooth with NoAtt received the least amount of vertical force. When the tooth was rotated distally, most attachments again received an intrusive force, while the 3Shape attachment displayed an extrusive force of 52cN. The NoAtt tooth received the largest intrusive force of 137cN, whereas the least force was applied to the HRecR and HEllipsoid, and this was extrusive and intrusive, respectively.

**FIGURE 5:**
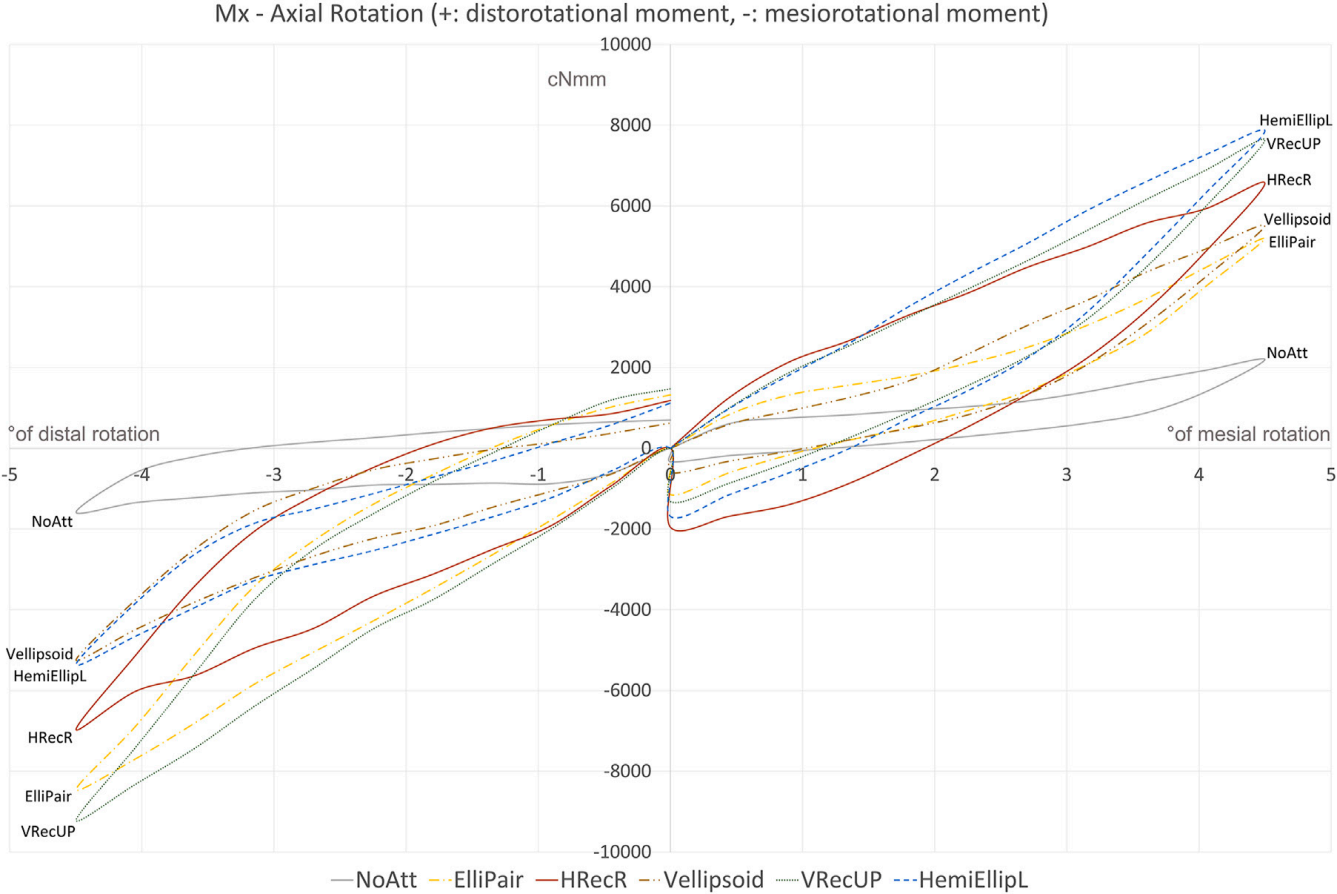
Best performing attachment from each of the five groups and the “NoAtt” setup. Vertical Rectangular attachments performed best on both mesial and distal rotations. HemiEllipL performed best on disto-rotation but not on mesio-rotation. Worth noticing the high moments created after only 1° of rotation and also the small degree of permanent deformation and fatigue in the aligner when looking at the de-activation part of the hysteresis loop. The time needed to reach 4.5° rotation either side (0°–4.5°) was 64°s, time that clinically translates to an instance when snapping the aligner in place.

When it comes to the moment created around the *X*-axis (derotation moment), for the mesially rotated tooth, the largest moments were encountered when the HemiEllipL was tested, followed by the vertical rectangular attachments of Group 1, VRecUP and VRecDOWN. Similarly, for the distal rotation, the largest moments were observed for the vertical rectangular attachments (VRecUP and VRecDOWN). For both mesial and distal rotation, the tooth with no attachment exhibited the least de-rotating moment (Figure 5).

### *y*-Axis

In the *y*-axis, when the tooth was rotated mesially 4.5°, the largest amount of force (73 cN), distal in this case, was when the VEllipsoid attachment was used and the least (5 cN) when the VRecDOWN was used, although this force had a mesial direction. For the distal rotation, all teeth received a mesial force with the VRecDOWN exhibiting the highest amount, at 216cN and the NoAtt setup the smallest, with around 10 cN.

Regarding the moment created around the *Y*-axis, when the tooth was rotated mesially, all setups received a buccal root torque with the highest seen with the VRecUP attachment and the smallest with the Bevelled. In the other direction, for the distally rotated tooth, a moment of lingual root torque was observed in all setups. The highest (7,704 cNmm) was seen in the VRecDOWN setup, whereas the Hellipsoid and NoAtt setups exhibited the lowest moments.

### *z*-Axis

Regarding the *z*-axis, again the vertical rectangular attachments (VRecUP and VRecDOWN) received the most amount of force. This had a lingual direction when the tooth was rotated mesial and was smallest in the Bevelled and in the NoAtt setup, whereas for the distally rotated tooth, this was directed buccally and was again smallest in the NoAtt tooth at around 60cN.

Regarding the moments, for the mesial rotation, the Bevelled attachment exhibited the smallest amount of moment (distal tipping), whereas the highest was encountered in the HemiEllipL and VRecUp setups. Finally, for the distal rotation, the highest moment (mesial tipping) was seen in the VRecDOWN setup (5,402 cNmm) and the lowest in the NoAtt setup (96 cNmm).

## Discussion

The results clearly demonstrate the variability between the different shapes and types of attachments used in aligner treatment. In the present study, there is a great range of moments, in all three axes of orientation and especially in the *x*-axis (mesio-/disto-rotation) of around 8,000 cNmm. Most importantly, this shows that a rotation of 4.5° in the aligner creates high moments at a level that most likely exceeds the moments needed for effective derotation. Although we do not know what the success criteria for the attachments are, and given that it is hard to find strict values to compare to in the literature, according to Proffit, the optimal force level for derotation was reported to be in the range 35–60 cN (Proffit et al., 2013). In addition, it is known that a challenging movement like lower molar uprighting requires moments in the range 1,200–1,800 cNmm (Raveli et al., 2017; Viecilli et al., 2009). Subsequently, these high moments could increase the risk of root resorption (Roscoe et al., 2015; Theodorou et al., 2019). The setup used in the present study reveals that already at 1° of mesial or distal rotation, the moments created should suffice for achieving an effective derotation (Figure 5). Therefore, it would make sense to not exceed 1° or 1.5° of derotation per step in aligner staging. Besides, it has been shown that the efficacy of tooth movement decreases significantly with a staging larger than 1.5°/aligner (Simon et al., 2014). This is probably the reason why Invisalign^®^ decided that the derotation provided by one stage never exceeds 2° (Align Technology, 2015).

In addition, in the absence of occlusal forces, our setup was “forgiving” as the aligners could be pushed in the occlusal direction and thus slightly disengage. One would assume that in the presence of function, the occlusal forces re-engage the aligner into position and the moments and forces created could potentially reach even higher levels. On the other hand, occlusion is happening only for a fraction of the 24 h (Proffit et al., 2013). Nevertheless, in a study with a comparable setup, but with an aligner-retention device where the aligner engagement was secured throughout testing, the rotational moments at 5° were smaller (Elkholy et al., 2019).

### Derotation moment, M_x_

If we focus purely on the amount of moment created to derotate the tooth (M_x_), our results show that the vertical rectangular attachments outperformed the others by creating the largest moments when the tooth was rotated either mesial or distal. This trend was seen in the smaller rotations of 1°, 2°, or 3° as well. To explain this, we should look at the design of these attachments where there is a large mesial and distal “flat” surface (3.5 × 1 mm), where the aligner can apply the needed derotational force. Also, it seems like the placement of the bevelled end of the vertical rectangular attachment (either up or down) does not play a significant role as both VRecUP and VRecDOWN created moments at comparable levels. Interestingly, the HemiElliL attachment created the highest moment but only when the tooth was rotated mesial (disto-rotation). This means that the flat surface of the attachment (located mesial) was more efficient in creating the needed force system to derotate the tooth compared to the bevelled end when the tooth was rotated distal. It seems that the flat end of the attachment works better as an active surface where a force is more easily applied.

Furthermore, our NoAtt setup exhibited the least amount of derotational moment (M_x_) for both mesial and distal rotations. These results demonstrate the need for auxiliaries like attachments in order to perform certain orthodontic tooth movements. This need has been previously reported for certain kinds of movement, as for the extrusion of an upper central incisor or derotation of a lower canine (Bowman, 2017; Elkholy et al., 2019; Savignano et al., 2019). Our results come in agreement with this, which most likely is due to lack of proper “grip” of the tooth by the aligner. It does make sense to think that attempting to reliably rotate a “round” tooth like a premolar requires some sort of a “handle” (Bowman, 2017; Cortona et al., 2020).

It was also interesting to observe that the ElliPair generated almost twice as much derotational moment, when the tooth was rotated distal compared to the opposite direction. To explain this, we need to consider the attachment’s design which consists of two hemi-elliptical shapes, the distal one positioned in the occlusal third of the crown, and the mesial one in the gingival third (Figure 4). It seems that during mesio-rotation, the attachment positioned more occlusal (here the distal one) is able to generate almost twice the amount of moment compared to the other. This most likely occurs because the aligner itself is much stiffer towards the occlusal end compared to the gingival (free) end and it probably “grips” the tooth better. Ultimately, this could mean that attachments placed in the occlusal third of the crown could be more efficient in providing much needed aligner retention. Nonetheless, this hypothesis has been questioned (Jones et al., 2009).

### Side Effects in the Three Planes

**FIGURE 6:**
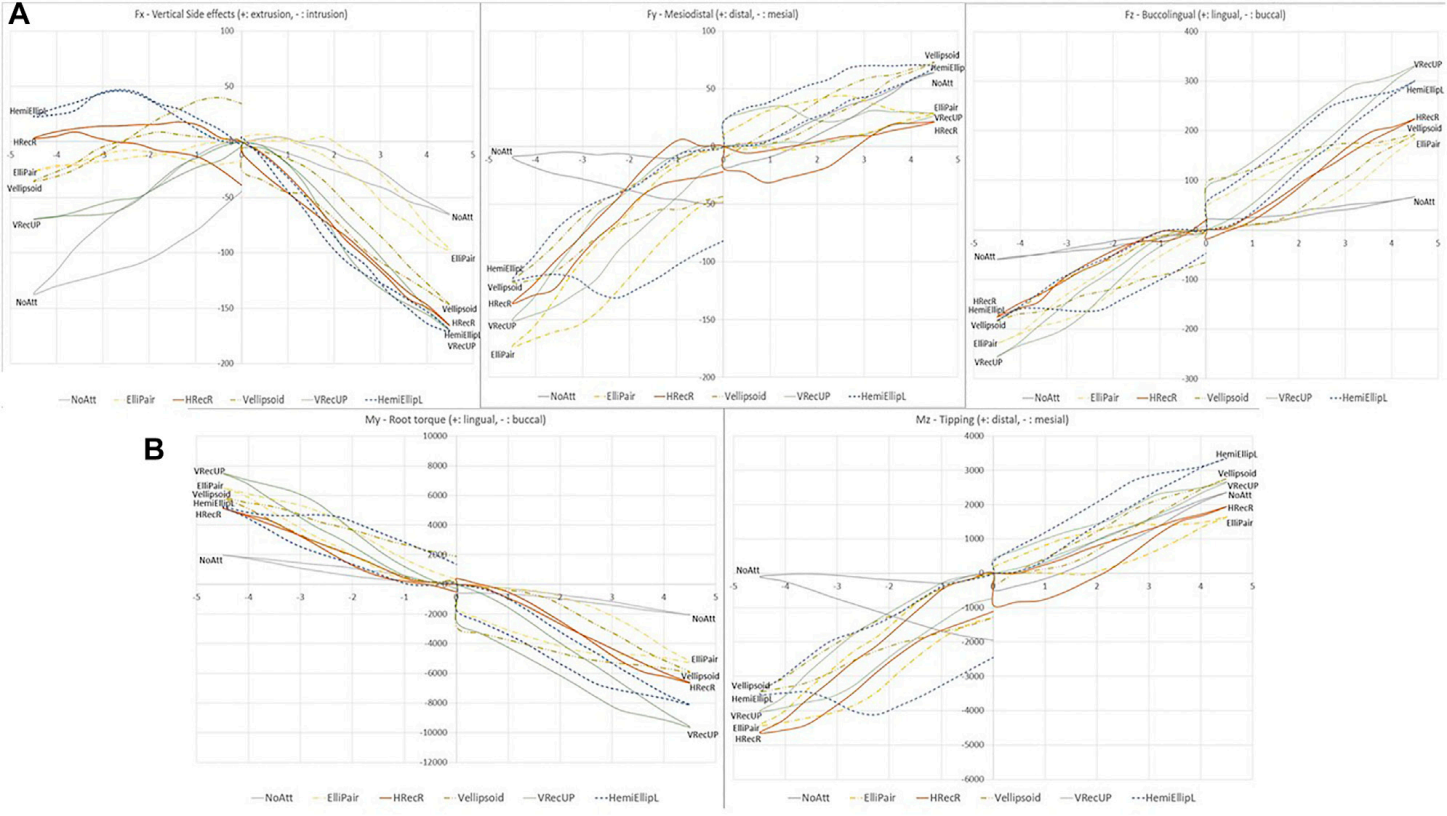
Side effects observed in all three planes of space for the best performing attachments of each group in terms of **(A)**. Forces and **(B)**. Moments. Note that the NoAtt setup exhibits the least amount of side effects in most charts.

A common side effect we encountered in all the attachment setups was the vertical forces created along the long axis of the tooth (*x*-axis), which in almost all occasions were intrusive (Figure 6). When the tooth was rotated mesially, all setups showed an intrusive force in a quite substantial amount which in most surpassed 100cN, with the highest in the 3Shape^®^ attachment. This far exceeds the recommended level of force needed for intrusion of 10–20 cN (Proffit et al., 2013), which of course raises questions of higher risk of root resorption. To explain this, one has to look into the biomechanics related to the premolar crown form. Due to the convexity of the crown, the force delivered to the tooth by the aligner when analyzed into the three-axial coordinate system almost always comes with an intrusive component which seems to be independent of any attachment used. This phenomenon has been well-demonstrated by Hahn et al. (Hahn et al., 2010) and is also known as the “watermelon seed” effect (Brezniak, 2008).

Interestingly, when the tooth was rotated distally, a few attachments demonstrated an extrusive force as side effect. This probably has to do with the shape of the tooth we used, and with the *in vitro* nature of our setup: in the absence of an occlusal forces aligner, re-engagement does not occur. Therefore, opposite forces can appear if the attachment is disengaged.

The vertical rectangular attachments also showed the highest moments in the *Y*-axis (mesio-distal). During the distal derotation, this was expressed as buccal root torque and during mesial derotation as lingual root torque, for all attachment types. Firstly, the non-symmetric crown form is to blame for this which does not allow for perfect rotation around the long axis. Secondly, it seems like due to the aforementioned crown anatomy, the aligner can “slide” more easily in the lingual surface, whereas the buccal surface is gripped better through the attachment. This could mean that the presence of a lingual attachment could minimize this type of side effect. Whether this side effect could be seen clinically, however, is a different matter; we know that one of the most challenging movements to achieve with aligners is root movement (Baldwin et al., 2008; Brezniak, 2008; Gomez et al., 2015). Therefore, torque overcorrections are often advised (Simon et al., 2014). It is probably due to this aligners’ inaccuracy that we do not see much lingual or buccal root torque when derotating a tooth. Finally, in the *y*-axis, just like in the *x*-axis, the NoAtt setup demonstrated moments in the lowest end of the range showing that a tooth with no attachment has the least severe side effects, but this has to be seen in the light of the reduced desired effect.

Moreover, all attachment types received a distal force during disto-rotation and a mesial force during mesio-rotation. Tipping is considered an “easy” and fairly predictable movement for aligners (Brezniak, 2008; Lombardo et al., 2017), and in contrast to what is valid for the root movement, this side effect could more easily become a clinical reality. This is important in extraction cases where both bodily movement and derotation are needed as it could increase the tipping of the tooth in the extraction space. This is crucial especially for the lower teeth as tipping seems to be more frequently seen in the mandible (Baldwin et al., 2008), due to the presence of a thicker cortical bone which makes bone resorption and thus translation more challenging.

Finally, when it comes to the *z*-axis (bucco-lingual), again, Group 1 VRecUP and VRecDOWN attachments exhibited the highest forces of more than 250cN (lingual during disto-rotation and buccal during mesio-rotation). This is probably related to the aforementioned premolar crown anatomy, which is not a perfect cylinder, but also to the attachment itself; it seems like the vertical rectangular attachments offer a much better “grip”.

The study comes with some limitations. The first one is of course the *in vitro* nature of the study itself. Despite the fact that we tried to replicate the oral wet conditions by lubricating the inside of the aligners with artificial saliva, there is an absence of occlusal forces, which might have an impact. Nevertheless, there are clinical implications in this investigation that a clinician orthodontist may find useful as clear aligners, whether produced in-house or not, have become a very popular treatment modality. In addition, it might be difficult to generalize the results of this study to other teeth than premolars, purely due to the difference in the dental anatomy. Nonetheless, the main principles probably apply to other teeth as well. Moreover, the material we have used is not the actual proprietary type of material used by Invisalign^®^ (SmartTrack): the first reason was it was not feasible to use it, then that we wanted to have the same setting for testing all the different setups. Last, at the time the experimental part was carried on, we did not have access to the new SmartForce optimized attachments currently used from Invisalign^®^.

## Conclusion

• Attachments should be considered necessary, at least for derotations of more rounded teeth like premolars.
• It seems that rotations above 1° generate moments, which are too high from a clinical point of view. Therefore, aligner steps of no more than 1–1.5° should be recommended for effective derotation of a premolar.
• The vertical rectangular attachments, due to their large flat active surface, perform best when derotating a premolar, but receive the most side effects in terms of tipping, torque, and intrusive force.
• Derotation of a premolar without any attachment was less efficient, despite showing the least side effects,

## Data Availability

The raw data supporting the conclusion of this article will be made available by the authors, without undue reservation.
